# Children with mixed developmental language disorder have more insecure patterns of attachment

**DOI:** 10.1186/s40359-018-0268-6

**Published:** 2018-11-15

**Authors:** Adele Assous, Ayala Borghini, Maryse Levi-Rueff, Guy Rittori, Bérangère Rousselot-Pailley, Christelle Gosme, Franck Zigante, Bernard Golse, Bruno Falissard, Laurence Robel

**Affiliations:** 1 APHP Hospital Necker Enfants Malades, Department of Child and Adolescent Psychiatry, 149-162 rue de Sèvres, 75015 Paris, France; 2 UFR Etudes Psychanalytiques, University Paris Diderot, Sorbonne Paris Cité, CRPMS, 75013 Paris, France; 3 SUPEA Pedopsychiatrie de liaison, SUPEA, CHUV, 1011 Lausanne, Switzerland; 4 CHS Sainte Anne, Department of Child and Adolescent Psychiatry, UPPEA, 1 rue Cabanis, 75014 Paris, France; 5 PCPP, Paris Descartes University, USPC, Paris, France; 6 CESP, INSERM U1178, Paris-Descartes University, USPC, Paris, 75014 Paris, France

**Keywords:** Language disorders, Attachment, Children

## Abstract

**Background:**

Developmental Language disorders (DLD) are developmental disorders that can affect both expressive and receptive language. When severe and persistent, they are often associated with psychiatric comorbidities and poor social outcome. The development of language involves early parent-infant interactions. The quality of these interactions is reflected in the quality of the child’s attachment patterns.

We hypothesized that children with DLD are at greater risk of insecure attachment, making them more vulnerable to psychiatric comorbidities. Therefore, we investigated the patterns of attachment of children with expressive and mixed expressive- receptive DLD.

**Methods:**

Forty-six participants, from 4 years 6 months to 7 years 5 months old, 12 with expressive Specific Language Impairment (DLD), and 35 with mixed DLD, were recruited through our learning disorder clinic, and compared to 23 normally developing children aged 3 years and a half. The quality of attachment was measured using the Attachment Stories Completion Task (ASCT) developed by Bretherton.

**Results:**

Children with developmental mixed language disorders were significantly less secure and more disorganized than normally developing children.

**Conclusions:**

Investigating the quality of attachment in children with DLD in the early stages could be important to adapt therapeutic strategies and to improve their social and psychiatric outcomes later in life.

## Background

Developmental Language Disorders (DLD) are one of the most frequent causes of consultation in child psychiatry.

Their prevalence was estimated to be 7.56% in a recent survey on 12,398 children aged 4 to 5 in the United Kingdom, making them among the most common disorders in early childhood. They impact both the development and the affective life of children, and therefore are a major challenge for public health [1]. Indeed, adolescents with a preschool history of speech impairment have good psychiatric outcomes if their language delay had been resolved by age 5, whereas they have significant attention and social difficulties in adolescence if they still have language difficulties [2]. Different terminologies have been used to describe language impairment in children, focusing on different aspects of these disorders. Although the term Specific Language Impairment (SLI) has been the most frequently used in the scientific literature so far, terminology has been the subject of recent debates [3], leading to a change in both definition and terminology in the Diagnosis Statistical Manual (DSM 5) [4].

In the International Classification of Diseases (ICD10) as well as in the DSM IV-R, the definition of “Specific Disorder of Language Acquisition” focuses on the specific nature of the disorder, and a distinction is made between expressive (ELD) and mixed expressive-receptive (MLD) types of language impairment. (APA, 1994; WHO, 1992) [5].

In the DSM-5 [4], “Language Disorders” are included in the neurodevelopmental disorders category. The distinction between expressive and mixed types of language impairment has been removed, as has the difference between verbal and nonverbal intellectual skills; in addition, language disorders can be associated with other diagnoses, such as autism spectrum disorders. In both definitions, the diagnosis comes with certain exclusion criteria, such as neurological disorders, hearing impairment, or intellectual disability, and language disorder has a significant impact on the child’s global functioning.

Despite changes in definition and terminology, the clinical questions raised on the subject of children with language difficulties remain the same. How do children with major language difficulties develop their thought processes, and how do they learn and interact with others? Since children’s language develops in interaction with their parents, caregivers and peers, language disorders cannot be studied without considering the processes at play in language development. Geller and Foley [6] therefore underlined the need to incorporate mental health constructs such as the attachment theory into the study of communication disorders, and to work from a relationship-based perspective with children who are language-impaired.

Attachment theory was first developed by John Bowlby [7]. He defined attachment as an enduring emotional bond that an individual form with another person (1977). He developed the concept of working models as generalized expectations and beliefs about oneself, the world and relationships to others, based on the early experiences babies share with their caregivers. He described two main categories of attachment, secure and insecure. The insecure category includes three different subcategories: insecure-avoidant, insecure ambivalent and insecure disorganized [8]. Whereas secure attachment is associated with better emotional and cognitive development, insecure-disorganized attachment can later be associated with externalizing and internalizing symptoms and disrupt different areas of development [9].

What are the interactions between attachment and language development in children? According to Van IJzendoorn and collaborators [10], language development is stimulated in the context of a secure attachment relationship. This was confirmed by Murray and Yingling [11], who demonstrated the additive effects of attachment and home stimulation on language competence, in 24-months-old typically developing children, especially for receptive abilities.

In children with DLD, the links between attachment and language development have not been studied. Although DLD has been demonstrated to be a markedly genetic disorder, genetic effects are complex, and involve strong links between genetic factors and the environment [12]. Onnis [13] suggested new directions for research, in order to study how early verbal and non-verbal attachment practices on the part of caregivers may mediate the expression of human systems of language. The link between language delay and early interactions was studied by Holditch-Davis et al., in prematurely born children [14]. They showed that mothers of language-delayed prematurely born children provided less interactive stimulation than mothers of children with typical language skills, suggesting that their child’s poor comprehension discourages maternal involvement. Negative feedback of this sort could also be present between children with DLD and their mothers, and interfere with the construction of a secure attachment, resulting in the potentialization of genetic factors influencing the development of language. The relation between language development and attachment is not linear, but is rather part of a circular process that takes place in the early interaction between the child and his caregivers.

Both language difficulties and insecure attachment patterns could then contribute to the high prevalence of psychiatric disorders observed later in life [15]. Indeed, Snowling and collaborators [2], in 71 adolescents from 15 to 16 years old with a preschool history of speech impairment, showed that those who still had specific expressive difficulties exhibited significant attention problems, and those with receptive and expressive difficulties had significant social difficulties in adolescence, whereas children whose language delay had been resolved by age 5 and a half had good psychiatric outcomes. The frequent comorbidities between psychiatric symptoms and language impairment point to the need to place the entire spectrum of language disorders in an integrated framework [16], and to adapt therapeutic approaches to the needs of each child early enough to prevent adverse language or social outcomes.

Therefore, the aim of our study was to study the patterns of attachment in children with DLD. Our hypothesis was that insecure patterns of attachment are more frequent in children with language disorders and contribute to their high rates of psychiatric disorders and poor social outcome in later adolescence. In order to study the construction of attachment in children with language disorders, we chose the Attachment Stories Completed Task developed by Bretherton [17]. This test can be used in young children from 3 years of age, because they can complement their narratives with actions, therefore limiting the impact of language on the construction of the stories. We used the Q-sort scoring validated by Milkovitch [18] on a French-speaking control group. This measure provides a dimensional analysis of attachment and enables a quantitative as well as a qualitative approach. We investigated the attachment profiles of 46 children with expressive or mixed expressive-receptive language disorders in comparison with 23 normally developing children. Our hypothesis was that children with language disorders were more likely to exhibit insecure attachment patterns than normally developing children, especially when they had a mixed expressive-receptive language disorder.

## Methods

### Population

Forty-six children, 12 girls and 34 boys aged 4–9 years with developmental language disorders (DLD), 11 with ELD and 35 with MLD, were investigated. These participants were recruited among children referred to our in- and out-patient clinic for severe and persistent language impairments between January 2012 and January 2014. The children underwent a comprehensive diagnostic examination consisting of a review of developmental history and of psychiatric and school records, a neuropsychological examination, and a standardised language test. An ICD-10 diagnosis was established by consensus between a psychologist, a speech therapist, and a senior psychiatrist involved in the evaluation of the child. Participants were diagnosed with DLD if they met the relevant ICD-10 criteria after language, psychological and psychomotor evaluations.

Language evaluation consisted of standardized validated tests in French of expression and comprehension. Inclusion criteria were scores adjusted to two standard deviations below the mean on expressive language subtests for ELD, and scores adjusted to two standard deviations below the mean on both expressive and receptive subtests for MLD.

Psychological evaluation included cognitive and projective assessments. Intellectual functioning was investigated with the appropriate Wechsler Intelligence Scale WISC-IV or the WPPSI-III tests. Inclusion criteria were a significant difference between the “verbal” and “performance” subscale scores (above 1.5 SD) and a Performance Intellectual Quotient (PIQ) over 70. We used projective tests (CAT, scenotest) for the psychopathological assessment.

For the psychomotor evaluation, we used standardised validated tests (NP-MOT, see below) assessing global and fine motor skills and coordination (Batterie d’évaluation des fonctions neuro-psychomotrices de l’enfant, NP-MOT, Vaivre-Douret L, ECPA, Paris, 2006).

Exclusion criteria were children with autism spectrum disorders, intellectual disability, neurological disorders or hearing loss. They were excluded after clinical and paramedical assessments (psychiatric evaluation, electroencephalography, audiometry).

The control group included 23 children, 15 girls and 8 boys who were recruited from the general population during their first months of life. This control group was part of a longitudinal study by the Lausanne research group and was chosen because of the absence of any language impairment. Ethics approval (N°20,110,508) was provided by the Ile de France ethics committee “Comité de Protection des Personnes” CPP-IDF2 de France II and written informed consent was obtained from participating parents and from the children when possible. Concerning the control group, the university of Lausanne ethics committee approved the research protocol.

### Language assessment

Different aspects of language were assessed using validated tasks in French from different language batteries (ELO, NEEL, see below) according to the possibilities and the age of the children: Receptive Vocabulary, Expressive Vocabulary, Word Repetition, phonology, Sentence Understanding, and Sentence Completion (assessment of oral language - *Evaluation du Langage Oral* - ELO, Khomsy, 2001; new tests for language assessment - *Nouvelles Epreuves pour l’Examen du Langage* - N-EEL, Chevrie-Muller C and Plaza, 2001).

These tests were validated on 900 and 540 French-speaking children respectively, aged from 3 to 11 and from 3.7 to 8.7 years. Results are presented as percentiles or standard deviations from the mean. For most participants, all tasks were administered in a 60-min session.

Since the scoring systems of these different tests differ, we adjusted the scoring system and determined severity levels, as previously described by Demouy et al. [19]. We first considered the means and standard deviations or percentiles for each task. To adjust the scoring systems to the different tests, for each participant we determined the corresponding age for each score, and then calculated the discrepancy between “verbal age” and chronological age. The difference was converted into a severity rating using a 5-point Likert-type scale, 0 standing for the expected level for chronological age, 1 for 1-year delay from the expected level for the chronological age, 2 for a 2-year delay, 3 for a 3-year delay, and 4 for more than 3 years’ delay. The expressive index was obtained by the summing of expressive vocabulary and sentence completion scores, and the receptive index by the summing of the receptive vocabulary and sentence understanding scores (Table 2). These three severity indexes were then used for the correlation analyses.

### Attachment story completion task (ASCT)

The ASCT has been specifically developped to assess attachment in children aged 3 to 8 [17]. Findings obtained with the ASCT have been validated in several studies with 3-year-olds, older preschoolers and school age children in several countries, including France [20]. Correlations have been reported with maternal AAIs, children’s self-representations and social competence at school. The ASCT has also been used in clinical group of children, such as children with cleft lip and/or palate in a recent longitudinal study [21].

It is composed of stories where the themes are intended to trigger the children’s attachment system and assess their attachment patterns. To complete the stories initiated, the children are given a set of dolls, each initially introduced as a member of a family (mother, father, children, and grandmother).

Each story beginning is presented by the examiner in a staged manner and the children are then asked to show and say what happens next.

There are 5 stories:

- Spilt juice: members of the family are together to celebrate the child’s birthday. Suddenly, the child spills some juice. What happens next?
- Hurt knee: the family goes into the garden. The child wants to climb on the rocks but his mother is worried and tells him that she is anxious that he might fall and hurt his knee. What happens next?
- Monster in the bedroom: The parents put their child to bed after dinner. The child plays in his room and hears a noise. The child says: “Oh no! There is a monster in my bedroom”. What happens next?
- Going away: The parents tell their children that they will be away for the week-end, and that they must stay with their grand-mother. What happens next? The examiner then provokes the departure of the parent’s figures if the participant does not. What happens during the parents’ absence and when they return?
- Reunion: The child wants to play with his dog Toby, with his mother’s agreement. However, Toby is not there. What happens next?

All the stories involve attachment-related issues. Indeed, the conflicts arising at the beginning of each story enable us to investigate how the children relate to parental figures.

Each assessment was filmed and then coded according to the *Attachment Story Completion Task Q-sort* (ASCT Q-sort) [18, 22].

The ASCT Q-Sort is composed of 65 items that describe the form and the content of the stories. This enables the quality of attachment of each participant to be described according to four categories: *security*, *disorganization (disruption)*, *deactivation* (*avoidance*), and *hyper activation* (*resistance-ambivalence*).

- *Secure* strategies are characterized by the ability to solve different conflicts with the help of parental figures.

-*Deactivated* attachment strategies tend to avoid conflicts; in the stories, parental characters are neither reassuring nor punitive.

- *Hyper-activated* strategies tend to focus on negative information, without being able to find a constructive solution.

- *Disorganized* narratives are characterized by the absence of a coherent strategy. For instance, the child loses control or is completely inhibited during play. The deactivated, hyper-activated and disorganized categories are defined as insecure [18].

The result of the test gives a description of the child’s quality of attachment in a dimensional manner (score for each category). In the development of the scoring system, the scores were normalized (T scores: M = 50, SD =10) on a control group of 187 French-speaking normally developing children [18]. Each child has a score on each of the four attachment style dimensions. Scores are significantly different from the mean when they are below 45 or over 55. However, a global attachment category can be deduced using the dimension where the participant scored the highest, or over 55. The results also enable an analysis of content and narrative characteristics according to 7 different scales: collaboration, parental support, positive narrative, expression of affects, reaction to separation, symbolic distance and poor narrative skills.

### Statistical analysis

Statistical analyses were performed on R software version 2.4.

We first investigated whether there was a correlation between the attachment patterns and the language severity index scores by calculating the Spearman correlation coefficients for the 4 attachment scores and the expressive and receptive severity indexes. We checked that the language scores within-groups did not correlate with attachment scores.

We used ANOVA to compare the characteristics of the children in the three groups (ELD, MLD, and control, *p* < =0.05).

A two by three contingency table with χ^2^ tests was used to compare attachment categories (secure versus insecure) and groups (MLD, ELD and control). We then performed multiple ANOVA followed by Tukey post hoc comparisons across the 4 attachment categories in the 3 groups, and across the 7 narrative scales for the three groups (*p* < =0.05).

## Results

### Characteristics of the groups

The characteristics of the children in the three groups are shown in Table 1. Children with DLD and children in the control group were significantly different for gender (X2(*n* = 69) =8.865, *p* = 0.03), age (*p* = 0.001), and VIQ scores (F (2,49) =75.92, *p* < 0.01), but not PIQ scores (F (2,54) =3.13, *p* = 0.05). Mean SES (socio economical status) was calculated as a mean of the levels of education and employment of mothers and fathers, as in Miljkovitch et al. (2003). There were no significant differences between the socio-economic status of the controls (2.91 (0.6)) and DLD group (2.59 (0.83)) (F (1,67) = 2.65, *p* = 0.1). Table 1.

**Table 1:** Characteristics of the children with expressive (ELD), mixed language disorder (MLD), and normally developing children

Group		ELD	MLD	Control
Number of children N (% of total)		11 (24)	35 (76)	23 (100)
Age (year. months) Mean (SD)		5.8 (0.99)	6.5 (2.16)	3.7 (0.07)
Gender	Male	8 (72)	26 (74)	8 (35)
n (%)	female	3 (28)	9 (25)	15 (65)
VIQ Mean (SD)		85 (23)	64 (14)	120 (27)
PIQ Mean (SD)		109 (14)	97 (16)	107 (55)
Expressive severity index Mean (SD)		2.45 (1.49)	4.15 (2.17)	-
Receptive severity index Mean (SD)		0.68 (0.68)	4.15 (2.09)	-

*ELD* Expressive Language Disorder, *MLD* Mixed language disorder, *VIQ* Verbal Intellectual Quotient, *PIQ* Performance Intellectual Quotient, *SD* Standard Deviation

### Correlation between attachment scores and language impairment scores

To check that the results of the ASCT were not biased by poor language understanding or expression, we investigated whether there was a correlation between the attachment patterns and the language severity index scores.

The Spearman correlation coefficients for the 4 attachment scores and the expressive and receptive severity indexes showed no correlation between the attachment scores and the two severity indexes (Table 2).

**Table 2:** Correlation between the 4 attachment dimensions and the expressive and receptive severity index in the Developmental Language Disorder (DLD) group

Attachment	Expressive severity index		Receptive severity index	
	Rho	*p*	Rho	*p*
Secure	−0.21	0.15	−0.19	0.19
Deactivated	0.19	0.19	0.21	0.15
Hyperactivated	0.002	0.98	0.06	0.65
Disorganized	0.11	0.44	−0.06	0.67

*Rho* Spearman correlation coefficient; *p* < =0,05

The children all attempted to recount what happened next, as requested. The stories completed by the children were both played out and put into words. The quality of the language was not taken into consideration in the coding system. We noticed that children with language impairments were looking for the reactions of the investigator during play.

### Children with DLD are more insecure than controls. Among children with DLD, those with MLD are more insecure and more disorganized

Differences in the proportions of insecure (deactivated, hyperactivated or disorganized categories of attachment) and secure attachment patterns across MLD, ELD, and control groups were investigated first.

The χ^2^ comparison showed that the proportion of children with insecure attachment was significantly higher in the group of children with a mixed language disorder (X2(*n* = 69) =7.914, *p* = 0.02) (Table 3).

**Table 3:** Proportion of secure and insecure attachment in children with ELD, MLD and control children

Attachment	ELD *n* = 11	MLD *n* = 35	Control *n* = 23	value	df	*p*
Secure (%)	0.63	0.25	0.56	7.91	2	0.019*
Insecure (%)	0.36	0.75*	0.43			

*ELD* Expressive Language Disorder, *MLD* Mixed Language Disorder, *C* Control

Pearson Chi-Square * < =0.05

We then looked which attachment dimensions differed between MLD, ELD, and control groups.

ANOVA comparisons showed significant differences for the secure and disorganized dimensions (Table 4). Post-hoc Tukey comparisons showed that children in the MLD group were significantly different from children in the control group for both the secure (*t* = − 7.63(3.08), *p* = 0.04) and the disorganized dimensions (*t* = 4.48(3.14), *p* = 0.05).

**Table 4:** Comparisons of the mean scores of attachment categories in ELD, MLD, and controls

Attachment Mean (SD)	ELD ‘= 11	MLD *N* = 35	Control *N* = 23	df	F	*p*
Secure	48.70 (11.68)	41.38 (11.47)	49.01(11.47)	68	3.71	0.03*
Deactivated	49.88 (10.12)	56.65 (10.56)	51.37(11.06)	68	2.59	0.08
Hyperactivated	50.57(10.13)	49.94(7.39)	48.72(10.55)	68	0.19	0.82
Disorganized	55.39(12.31)	58.28(10.62)	50.79(13)	68	2.82	0.05*

ANOVA *: *p < =0.05*

### Children with MLD, but not ELD, have poorer narrative skills and express fewer affects than controls

The ANOVA comparison of the scores obtained on the 7 different narration scales by the three groups of children showed significant differences between groups in the expression of affect and poor narrative skill dimensions (Table 5). Tukey Post-hoc comparisons showed a significant decrease in the expression of affect in the MLD group in comparison to both the ELD group (*t* = − 12.88(4.43); *p* = 0.014) and the controls (*t* = − 8.76(3.44); *p* = 0.035), as well as poorer narrative skills in the MLD group, compared to controls (*t* = 8.18(3.4); *p* = 0.031).

**Table 5:** Comparison of narrative scales in ELD, MLD, and controls

Attachment Mean (SD)	ELD *N* = 11	MLD *N* = 35	Control *N* = 23	df	F	*p*
Collaboration	54.04 (12.15)	45.85 (11.79)	50.44 (12.14)	68	0.71	0.4
Parental Support	48.18 (11.49)	46.69 (9.54)	48.48 (11.51)	68	0.28	0.59
Positive narrative	45.77 (16.70)	41.45 (11.13)	48.82 (12.41)	68	3.91	0.05*
Expression of affect	52.18 (13.75)	39.29*(14.19)	48.05 (9.87)	68	2.71	0.10
Reaction to separation	50.55 (11.15)	44.86 (9.12)	48.01 (8.57)	68	0.55	0.46
Symbolic distance	55.37 (9.31)	48.40 (9.77)	49.43 (12.45)	68	0.05	0.8
Poor narrative skills	51.71 (11.52)	59.65*(12.78)	51.46 (10.00)	68	4.2	0.04*

ANOVA comparison. *p < = 0.05

The differences seen in the expression of affects and the poor narrative skills could be related to the language impairment among children with MLD, since we found a weak correlation between the severity score on the expressive scale and the narrative scales “Symbolic distance” (Rho = − 0.4; *p* = 0.01) “poor narrative skills” (Rho = 0.35; *p* = 0.03), and “Appropriate Expression of Affect” (Rho = 0.34; *p* = 0.04) in the MLD group, but not in the ELD group (Spearman correlation coefficient; *p* < =0.05).

## Discussion

To our knowledge, this is the first time that quality of attachment has been assessed in children with language disorders or specific language impairment using the ASCT. Results showed that the attachment style in children with mixed language disorders (MLD) was less secure and more disorganized than in normally developing children.

The children included in this study had severe and persistent language disorders despite speech remediation. Our results show that it is possible to assess their attachment patterns with the ASCT despite their language impairment. The children were able to continue the story initiated by the investigator using dolls, acting and language. Moreover, we showed that the results we obtained on the patterns of attachment were not influenced by the children’s difficulties in expression or understanding, since attachment scores in the four categories were not correlated to the expressive, receptive, and global index severity scores.

Our results show that children with mixed language disorders have significantly lower scores on the secure dimension and higher scores on the disorganized dimension, than children in the control group. This is not the case for children with expressive language disorders. The children in the three groups were able to perceive theme, but the children of the MLD group experienced greater difficulty in expressing their affects and in elaborating coherent stories.

Qualitatively, disorganization was manifested through several aspects: children lost their symbolic distance by acting themselves instead of acting through the dolls, they denied separation by erasing the beginning of the story, they launched into catastrophic never-ending scenarios, with very little cooperation between the different dolls and poor support from the parental figures. This disorganization was clearly revealed by the themes of separation and conflict contained in the ASCT, since the same children developed very restrictive scenarios in their free play (scenotest).

Higher disorganization scores among children of the MLD group could reflect the impact of the child’s poor comprehension on his caregiver’s involvement, which would then interfere with the construction of a secure attachment, as shown in prematurely born children with language delay [13]. Indeed, the understanding of language precedes its expression, and is stimulated in the context of secure attachment in normally developing children [10]. In response to their child’s poor understanding, parents may provide less verbal and non-verbal stimulation, and anticipate their children’s needs. The need for the parents to adapt to their child’s speech difficulties in turn increases the child’s linguistic and affective dependence [23]. This dependence is illustrated in the ASCT task by the fact that children are very dependent on the reactions of the investigator. The experience of separation, which is necessary for language to develop [24], becomes more and more difficult to overcome, and the process of separation more difficult to complete. As an aggravating factor, the difficulty these children have in using language to express their feelings and to build relationships with others interferes with the “linguistic co-construction of internal coherence” [25].

When the understanding of language was not impaired, in the ELD group, the children were as secure as in the control group, suggesting the early, central role of understanding in the co-construction of a secure attachment. However, we must underline that not all the children in the MLD group were disorganized. This suggests that attachment disorganization is not the linear consequence of difficulties in understanding, but rather results from a circular process that takes place in the early interaction between the child and his caregivers.

The disorganized patterns of attachment observed in children with mixed DLD could be related to the high prevalence of psychiatric disorders and to the poor social prognosis described in these children. Indeed, Yew and O’Kearney [26] in their systematic review and meta-analysis reported a high prevalence of psychiatric comorbidities with a marked increase in the severity of diverse emotional, behavioural and ADHD symptoms in DLD children. Adolescents with a history of DLD report levels of peer problems that are 12 times higher than for those without problems, and they are less emotionally engaged in close relationships [27, 28]. Finally, children with mixed DLD have the poorest social prognosis [15, 29]. The relationship between insecure attachments and psychopathology has already been demonstrated [9, 30, 31]. We therefore think that it may be very important to investigate the attachment patterns of children with DLD early on, together with language and cognitive assessments. The need to investigate additional factors has already been underlined by the Catalise consortium (a multinational and multidisciplinary Delphi consensus study of problems of language development) who recently proposed a set of consensual statements aiming to refer and assess children with language disorders [32]. These factors need to be evaluated early on, in order to improve the developmental trajectory of these children, and to decrease the serious negative consequences of their disorder for their educational and social outcomes [33, 34].

We have shown here that the ASCT can be used to investigate the attachment representations of children with language disorders. The initiation of the stories by the investigator helps the children to construct their scenarios, and the use of dolls enables them to unroll their stories even if words or syntax are lacking. The playful dimension of the task removes the stress of the evaluation, both for the child and for his/her parents. The information included in the test can be explained to the parents and can facilitate their understanding of the psychological difficulties encountered by their child, and the need for a psychotherapeutic approach combined with speech remediation when needed. In addition, the Q-score coding system developed by Miljkovitch [18, 22] gives a description of the attachment profile of each child according to a continuum, in a dimensional rather than in a categorical perspective, and gives access to the content of the stories. It is also sensitive to the changes induced by therapeutic approaches [35].

## Limitations

There are several limitations to our study.

A first limitation is related to the fact that we have investigated the children’s patterns of attachment with the ASCT, a test using language, in a group of children with a language impairment. This is the reason why we have checked through the correlation tests that there was no correlation between the scores of language impairment and the results on the category of attachment. ASCT has been specifically developped to assess attachment in children aged 3 to 8, and it has already been used in children with cleft lip and/or palate in a longitudinal study [20]. Other instruments for the evaluation of children’s attachment through parental or professional reports have been developed, such as a questionnaire aimed to measure attachment of three to 6 year old children by observers in kindergarten, but the results obtained were not concordant with the other attachment measures, such as the strange situation for preschool children and the attachment story completion task [36]. Parent report on their child’s attachment profile have been developed only for very young children under 1 year [37]. Therefore, the ASCT seems to be the best way to evaluate attachment representation in our population, despite language impairment.

A second limitation is related to the fact that our sample size is small, impacting the statistical power of our analysis. Indeed we could not make a power analysis to calculate the sample size, because severe language disorders are not frequent. Therefore we were not able to include more participants through our inpatient unit for children with language disorders. However, we obtained statistically significant differences between groups. Moreover, we have already published research papers comparing the characteristics of smaller groups on patients with DLD on multiple tasks [38].

A third limitation is related to the fact that the control group was recruited by another team, in another French-speaking country, with a different gender ratio and a smaller group of children. However, we checked that the two groups did not differ in terms of socio-economic status, and that there was no difference in the distribution of the 4 attachment categories according to gender (ANOVA, *p* > =0.05). We had the same results when comparing the ASCT scores of the DLD groups to the theoretical mean. Indeed ASCT scores were previously normed and validated on a large sample of typically developing children. Children in the two groups were significantly different according to their VIQ (*p* = 0.0001) but not to their PIQ (*p* > 0,05), as a consequence of the language impairment among children with DLD. However, the fact that the control group was younger reduced the differences between the DLD and control groups in their raw intellectual performances. Further to this, a previous study by Miljkovitch [20] showed that attachment profiles were not correlated to IQ, and we have shown here that there was no correlation between the severity of the language impairment and the distribution of the attachment patterns. On the contrary, we found a weak correlation between the severity scores on the expressive and narrative scales symbolic distance, poor narrative skills and appropriate expression of affects in the MLD group. The quantitative results obtained for narrative abilities on these three scales thus need to be interpreted with caution, as is the case with young pre-schoolers. Indeed, for pre-schoolers, Miljkovitch et al. [20] stated that the most important aspect is how the children process the attachment themes presented in the stories and how they respond to themes of distress, suggesting the need to “consider secondarily how these reactions could influence children’s narrative ability”. We think that the same precautions need to be taken with children presenting a language impairment.

## Conclusions

Our study is a first attempt to capture the vulnerability of children with DLD towards psychiatric disorders through the perspective of attachment. We found that the use of the ASCT was well suited to the characteristics of children with DLD, especially at younger ages, when therapeutic interventions are thought to be the most efficacious. Our results showed that children with MLD were more insecure than children in the general population, with a larger proportion of disorganized profiles. Since insecure attachment is associated with a higher risk of developing psychiatric disorders, we think that investigating the quality of attachment in children with DLD is useful, in order to adapt therapeutic interventions.

We need to confirm these results on a larger group of children, to see if the attachment profiles we have described are stable over time even if there is language improvement, and to ascertain whether insecure profiles are correlated with psychiatric disorders at later ages. The next step would be to investigate whether the combination of speech remediation and specific psychotherapeutic approaches has an impact on both the attachment patterns of these children, and their social and psychiatric outcomes.

## Abbreviations

ASCT: Attachment story completion task
DLD: Developmental language disorder
ELD: Expressive language disorder
ELO: Oral language assessment for children
ICD: International classification of diseases
MLD: Mixed expressive receptive language disorder
NEEL: New language assessment for children
SLI: Specific language impairment
WISC: Wechsler intelligence scale for children
WPPSI: Wechsler
